# Fighting to Train—Implementation of a *Train Like You Fight* Joint Role 2 Austere Surgical Care Curriculum

**DOI:** 10.1093/milmed/usaf576

**Published:** 2025-12-04

**Authors:** Matthew D Tadlock, Dan S Mosely, Jay B Baker, Sean Keenan, Ronald David Hardin, Tyson Erik Becker, Richard A Jarret, Jennifer M Gurney

**Affiliations:** Department of Surgery, Uniformed Services University of the Health Sciences, Naval Medical Center San Diego, San Diego, CA 92134, United States; Committee on Surgical Combat Casualty Care, Joint Trauma System, Joint Base San Antonia-For Sam, Houston, TX 78234, United States; Joint Trauma System, DoD Center of Excellence for Trauma, Joint Base San Antonio-Fort Sam, Houston, TX 78234, United States; Joint Trauma System, DoD Center of Excellence for Trauma, Joint Base San Antonio-Fort Sam, Houston, TX 78234, United States; Joint Trauma System, DoD Center of Excellence for Trauma, Joint Base San Antonio-Fort Sam, Houston, TX 78234, United States; University of Colorado School of Medicine, Anschutz Medical Campus, Aurora, CO 80045, United States; Committee on Surgical Combat Casualty Care, Joint Trauma System, Joint Base San Antonia-For Sam, Houston, TX 78234, United States; Brooke Army Medical Center, JBSA, 3551 Roger Brooke Dr, San Antonio, TX 78234, United States; Brooke Army Medical Center, JBSA, 3551 Roger Brooke Dr, San Antonio, TX 78234, United States; Joint Trauma System, DoD Center of Excellence for Trauma, Joint Base San Antonio-Fort Sam, Houston, TX 78234, United States; Committee on Surgical Combat Casualty Care, Joint Trauma System, Joint Base San Antonia-For Sam, Houston, TX 78234, United States; Joint Trauma System, DoD Center of Excellence for Trauma, Joint Base San Antonio-Fort Sam, Houston, TX 78234, United States

## Abstract

**Introduction:**

Each military service has independently developed austere resuscitative and surgical care (ARSC) team training courses that vary in use, content, duration, tactical exposure, and clinical emphasis. A 2020 Department of Defense Inspector General audit identified training deficits for ARSC teams. To close this gap, we developed ARSC curriculum standards for the Joint Force.

**Materials and Methods:**

A work group of Tri-Service appointed multidisciplinary subject matter experts (SMEs) reviewed available Role 2 and ARSC training courses to identify common curricular elements, best practices, and training gaps. Through an iterative process, these findings were incorporated into a standard curriculum organized into modules subdivided into terminal learning objectives (TLOs) and enabling learning objectives (ELOs), which were validated against Service doctrinal ARSC capability requirements.

**Results:**

Curricula from 12 different courses were identified and reviewed. Most prepared teams for Counter Insurgency operations characterized by short holding times and rapid aeromedical evacuation. Eighty-two clinical and nonclinical common curricular best practices were identified. Only four courses had 50% or more of SME-recommended curricular elements. Identified curricular gaps included prolonged holding (0% of courses), definitive austere surgical care (0%), austere critical care (25%), simulated tactical exposure (25%), night operations familiarization (37.5%), and simulated operational environment (62.5%). Ten modules were created comprised of 20 TLOs and 259 ELOs incorporating curricular best practices and identified gaps.

**Conclusion:**

Military medicine is preparing surgical teams for the war just fought, not the future fight and lacks a joint standard for training Role 2/ARSC surgical teams, which is a risk to force and to mission. We close this gap by creating the first Joint Role 2 forward surgical/ARSC curriculum.

## INTRODUCTION

The U.S. Department of War (DoW) is responsible for maintaining a global casualty response system. During wartime or contingency operations, severely injured casualties move along a continuum of battlefield care from point of injury (POI) through increasing levels of battlefield medical capabilities, then out of the theater of operations, and finally back to the United States for rehabilitation, convalescence, and hopefully return to duty. When an injured patient enters the civilian trauma system, what can be a rapid ambulance transport and a single elevator ride in a civilian trauma center could be hundreds of miles for a combat casualty and necessitate multiple helicopters and/or airplanes, and three or more different care teams in the military’s battlefield trauma system. The complexities of the DoW’s global casualty response system have training implications and impacts on all aspects of the battlefield care continuum which is organized into Roles of Care (Role 1 through Role 4). The military’s continuum of care has been studied and, in some cases, adopted by certain civilian humanitarian response constructs, and military lessons learned can be applied to humanitarian and disaster response events; therefore, understanding how to train these teams has implications beyond the military application of team training.^1^^,^^2^

The highest level of care on the battlefield is a “Role 3” (R3), commonly known as a Combat Support Hospital or a Theater Hospital. Definitive care, advanced surgical care, and specialty care is provided at these Role 3 Military Treatment Facilities (MTF).^3–5^

POI care is Role 1 or prehospital care. “Role 2” (R2) forward surgical care bridges the time/space gap between POI and R3 care and exists within a broad spectrum depending on the operational platform and the mission being supported. Contemporary R2 forward surgical teams, especially as they were deployed during the Global War on Terror (GWOT), employ damage control resuscitation (DCR) and damage control surgery (DCS) to stabilize severely injured casualties so they arrive alive to the R3.^3–7^

R2 surgical teams have evolved substantially during the course of GWOT operations in Iraq, Afghanistan, and Syria. Since 2001, each of the military services has created multiple constructs of R2 teams, each with varying size and capability.^8–10^ The primary function of these small surgical teams was to provide blood product resuscitation and DCS within the “golden hour.” As their use proliferated, these teams became smaller and smaller to meet the needs of the units they supported, which required more mobility and less weight/cube of personnel and equipment.^6^^,^^8–10^ No matter the team size or capability, their utilization was predicated on the following assumptions: (1) air superiority, (2) continuous and uninterrupted supply chains, (3) substantial medical evacuation (MEDEVAC) capabilities, and (4) an overall low level of casualties.^8^^,^^9^ This operational concept of smaller teams performing early surgical care close to the POI-saved lives during recent operations in the U.S. Central (CENTCOM) and Africa (AFRICOM) Combatant Command areas of responsibility.^11^^,^^12^

While not comprehensive, mostly because of the continuous evolution of these teams, **Table 1** demonstrates 13 different types of R2 surgical teams created by the medical components within each service to meet various mission demands over the last 25 years.^8^^,^^9^^,^^13^ Despite the huge investment into creating and deploying these doctrinal, non-doctrinal, and ad-hoc teams, there has not been an equivocal investment in training R2 surgical teams for mission success.^14–16^ This paucity of training generates not only a risk to the mission that they are supporting, but also risks the team’s ability to respond to the threats of the environment and to stay safe as well as mission capable. Given the military’s emphasis on “train like you fight,” ground force commanders likely assume that these small surgical teams are trained for the clinical, operational, and tactical aspects of the forward surgical mission; however, many times this in an incorrect assumption not often completely articulated to ground force commanders.

**Table 1.** Sample of Past and Present Role 2/Austere Resuscitative and Surgical Care Teams in U.S. Military*

Team Name	Acronym	Service	# Personnel	Type of Personnel	Estimated Mission Capacity	Operating Room (OR) Capacity	Intrinsic Evacuation Capability
**Role 2 Light Maneuver: A light and highly mobile medical unit able to conduct advanced resuscitation procedures, including damage control resuscitation (DCR) up to damage control surgery (DCS).^1^**							
Surgical resuscitation team	SRT	Joint	4-5	PA, GS, EM physician, CRNA, commo	2-6 DCS consecutively	1 OR table	Yes
Forward resuscitative surgical team	FRST	Army	10	GS, ortho, EM physician, CRNA, CCRN, EMRN, ST, LPN, 68W, MSC	2 DCS + 5 DCR/24 h 3-4 DCS + 8 DCR/72 h	1 OR table	No
Expeditionary resuscitative surgical team	ERST	Army	8	GS, ortho, EM physician, CC physician, CRNA, CCRN, EMRN, ST	1-2 DCS + 2-3 DCR	1 OR table	Yes
Special operations surgical team	SOST	Air Force	6	GS, EM physician, CRNA, CCRN, RT, ST	2-10 DCS	1 OR table	No
Tactical critical care evacuation team	TCCET	Air Force	3	CC, CCRN, RT	3 DCR	N/A	Yes
Tactical critical care evacuation team-enhanced	TCCET-E	Air Force	5	GS, EM physician, 2x CRNA, ST	3-5 DCS	1 OR table	Yes
Ground surgical team	GST	Air Force	6	GS, anesthesiologist, EM physician, CCRN, ST, MSC	3-5 DCS initial, 7-11 DCS extended	1 OR table	No
Damage control surgical team	DCST	Navy	7	GS, EM, CRNA, CCRN, EMRN, IDC, ST	2 DCS, 2-3 DCR	1 OR table	No
Expeditionary resuscitative surgical system	ERSS	Navy	7	GS, EM physician, CRNA, EMRN, PA, ST, RT	4 DCS, 6 DCR/72 h without resupply	1 OR table	No
**Role 2: “Advanced trauma management and emergency medical treatment including continuation of resuscitation started in Role 1.”** **Includes DCR, DCS.** ^1^							
Golden hour offset surgical team**	GHOST	Army	10	1-2 GS, 1-2 CRNA, ST, 1-2 RN, medic	2 DCS + 5 DCR/24 h; 3-4 DCS + 8 DCR/72 h	1-2 OR tables	No
Forward surgical team**	FST	Army	20	GS, Ortho, CRNA, OR tech, ER nurse, medics, LVN	10 OR cases in 24 hours, 72 hours of operations without resupply	2 OR table	No
Forward resuscitative surgical detachment***	FRSD	Army	20	3 GS, 1 ortho, 2 CRNA, 1 EMRN, 1 CCRN, 1 OR RN, 3 ST, 3 LPN, 4 68W, 1 MSC	4 DCS + 10DCR/24 h 6-8 DCS + 12 DCR/72 h	2 OR tables	No
Shock trauma platoon + forward resuscitative surgical platoon	STP/FRSS	USMC	∼24	GS, ortho, anesthesiologist, ST (2) CCRN, corpsman (13), PA, IDC (2), EM physician, EMRN	18 DCS/48 h without resupply	1 OR table	

* Adapted from Baker et al, Bingham et al, and Vasquez et al.^8^^,^^9^^,^^13^

** These teams were frequently split during the Global War on Terror.

*** Able to doctrinally split in half to provide R2LM capability.

PA, physician assistant; GS, General Surgeon, GS; EM, Emergency Medicine; CRNA, certified registered nurse anesthetist; commo, communications specialist; ortho, orthopedic surgeon; h, hour; EMRN, emergency medicine registered nurse; CCRN, critical care registered nurse; ST, surgical technician; LPN, licensed practical nurse; 68W, combat medic; MSC, medical service corps officer; CC, critical care; RT, respiratory therapist; IDC, independent duty corpsman; USMC, United States Marine Corps; R2LM, role 2 light maneuver; DCR, Damage Control Resuscitation; DCS, Damage Control Surgery.

The Joint Trauma System (JTS), the DoW reference body for trauma care, had a primary line of effort within in the Committee on Surgical Combat Casualty Care (CoSCCC) to mitigate risks of these small surgical teams, given that individual skills and team training are both crucial for mission success.^6^ Gaining traction to develop standardized team training proved to be a challenge despite the 2017 Joint Chiefs of Staff Requirements Oversight Council directing the creation of a curriculum for training small, mobile surgical teams that operate effectively in the austere environment.^17–19^ To support interoperability and clear communication through the medical community, the capability was defined as *Austere Resuscitative and Surgical Care* (ARSC): “advanced medical capability delivered by small teams with limited resources, often beyond traditional timelines of care, and bridges gaps in roles of care in order to enable forward military operations and mitigate risk to the force.”^8^^,^^10^

An existing course for these teams which had gained Joint Service popularity among surgical teams was allowed to sunset despite the above requirement and the findings from a DoW Inspector General (IG) audit of R2/ARSC deployments which reported extensive deficits in this training. The IG recommended improving “surgical and tactical training to better prepare mobile medical teams for… austere environments.”^18^ Multiple other documents directing the Medical Services to support training programs for R2 teams deployed to the joint forward surgical care environments were not actioned. The CoSCCC ARSC subcommittee and JTS ARSC curriculum work group were established to develop products, including a joint curriculum, to address the challenges of these forward-deployed surgical teams to help close the gaps that risked the effectiveness of these teams and battlefield survivability; both the survivability of casualties, as well as these teams.^8^ This article describes the methodology used to develop a standardized curriculum for the Joint Force that covers all aspects of the ARSC mission and forward surgical care.

## METHODS

### Task Analysis and Work Group Formation

The JTS analyzed the scope of the task and validated the end state: a modular Joint R2 forward surgical curriculum that focused on small surgical teams and the ARSC mission. The curriculum had to be tailored to the current, as well as the anticipated future, needs of diverse Service R2/ARSC surgical team capabilities. All courses in the Army, Navy, U.S. Marine Corps, and Air Force were catalogued and evaluated, and best practice curricular elements were incorporated into a cohesive corpus of standards that could be integrated into existing or future training courses.

To meet the requirements of the Defense Health Agency (DHA) and Service bureaucracies (given that a home-grown course that exceeded the 2017 Joint Requirements Oversight Council mandate was allowed to sunset), multiple administrative steps were taken to ensure eventual adoption of this course (appointments, charters, service selection of workgroup members, etc.) and an ARSC work group (WG) was established under the JTS. As directed by the charter, each Medical Service (Army, Navy, Marine Corps, Air Force) and the Special Operations Command appointed their subject matter experts (SMEs). SMEs were mandated to include trauma surgeons, clinicians with significant operational experience, international ARSC experts, other trauma SMEs in logistics and military capability development, medics, and nurses. The ARSC WG met bimonthly for 15 months.

### ARSC Course Analysis

All known existing courses with available curricula or course materials were identified, collated, and categorized, including international military courses. A comprehensive analysis of all curricular elements, materials, and course agendas was performed followed by a gap analysis. Current course curricula were compared to each other, to what was in the existing literature, the JTS Clinical Practice Guidelines (CPGs), and to six years of CoSCCC meeting minutes where the ARSC capability was discussed, debated, and refined, to include lessons learned from the Ukraine war.^20^ Through a methodical and iterative process (Box S1), the WG identified the essential and shared curricular elements to create best practices for the Joint R2/ARSC curriculum.

### Module Development

Essential and shared curricular elements were organized into Modules and then subdivided into terminal learning objectives (TLOs) and enabling learning objectives (ELOs) to comply with DoD curriculum standards. TLOs describe what learners should be able to do, and ELOs describe the knowledge or skills required to perform the TLO. Medical Service specific doctrinal ARSC team capability requirements were used to validate these modules to identify additional curricular gaps.

## RESULTS

**Figure 1.**
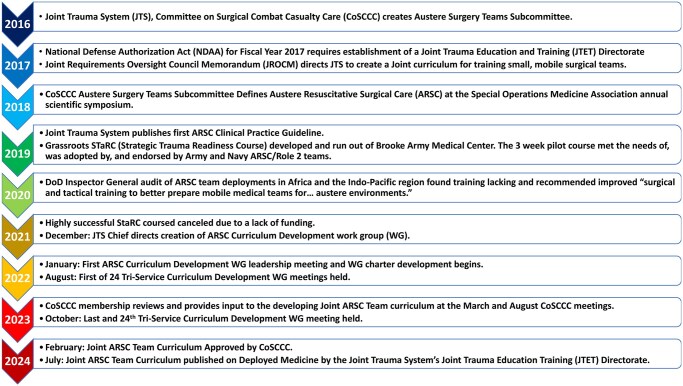
Austere resuscitative surgical care timeline.

**Figure 1** demonstrates a timeline of this comprehensive endeavor and **Table S1** demonstrates the identified courses whose curricula and course materials were used to develop Joint Force R2/ARSC curricular standards. The analysis identified 82 clinical and nonclinical common objectives. Both the Advanced Surgical Skills for Exposure in Trauma (ASSET+) and the Combat Orthopedic Trauma Course (COTS+)^21^^,^^22^ were included in this analysis because many individual ARSC courses incorporate either these courses or elements of them into their curricula.

**Table 2** compares eight service run team-based courses. ASSET+, COTS+, and the Definitive Surgical Trauma Care course were not included in this analysis because these courses are designed for the individual surgeon and not the entire team. A total of 24 curricular elements were identified and compared between the eight courses. Of the courses analyses, only the North Atlantic Treaty Organization (NATO) Special Operations Surgical Team (SOST) (50%), the Army Strategic Readiness Center (STaRC) (58.3%), the Air Force Ground Surgical Team (54.2%), and the Navy Expeditionary Resuscitative Surgical System (ERSS) (79.2%) courses included 50% or more of these 24 curricular elements. Seven (87.5%) courses routinely had appropriate SMEs facilitating scenarios, providing course quality control, and giving structured feedback. Only 50% of courses formally included an after-action review process after each simulated scenario or mission.

**Table 2.** Comparison of Curricular Elements of Team Based Austere Resuscitative Surgical Courses

Course characteristics	STaRC	ATTC	NTTC	Navy ERSS	USMC R2	S2T2	GST	NATO SOST	Total (%)
**Course design**									
# Days	21	15	16	10 (8)	2-5^*^	5	11	5	N/A
Didactic	Y	Y	Y	Y	N	Y	Y	Y	8 (100)
Simulation	Y	Y	Y	Y	Y	Y	Y	Y	8 (100)
Cadaver	Y	Y	Y	N	N	Y	Y	N	5 (62.5)
Live tissue	Y	Y	N	N	N	N	Y	N	3 (37.5)
Scenario based	Y	Y	Y	Y	Y	Y	Y	Y	8 (100)
Just in time hospital rotations	Y	Y	Y	N	N	N	N	N	3 (37.5)
SME feedback & quality control	Y	N	Y	Y	Y	Y	Y	Y	7 (87.5)
**Individual procedural skills**									
Equipment familiarization	Y	N	Y	Y	Y	Y	Y	Y	7 (87.5)
Non-surgeon procedural skills lab	Y	Y	Y	N	N	Y	N	N	4 (50)
**Operational/tactical focus**									
Simulated operational environment	Y	N	N	Y	Y	Y	N	Y	5 (62.5)
Tactical exposure	N	N	N	Y	N	N	Y	N	2 (25)
Vehicle training	N	N	N	Y	Y	Y	N	N	3 (37.5)
Noisy environments	N	N	N	Y	N	N	Y	Y	3 (37.5)
Maritime environment	N	N	N	Y	N	Y	N	N	2 (25)
Night operations	Y	N	N	Y	N	N	Y	N	3 (37.5)
Buildings of opportunity	N	N	Y	Y	N	N	Y	Y	4 (50)
Hasty field conditions	Y	N	N	Y	N	N	Y	Y	4 (50)
Interoperable operational scenarios	N	N	N	Y	N	N	N	Y	2 (25)
Incorporates AAR process	Y	N	Y	Y	N	N	N	Y	4 (50)
**Clinical focus**									
Trauma lane/TCCC refresh	Y	N	N	Y	N	N	N	N	2 (25)
Definitive surgery	N	N	N	N	N	N	N	N	0
DCR/DCS	Y	Y	Y	Y	Y	Y	Y	Y	8 (100)
Prolonged holding	N	N	N	Y	Y	N	N	N	2 (25)
Austere critical care	N	N	N	Y	Y	N	N	N	2 (25)
**TOTAL ELEMENTS (%)**	**14 (58.3)**	**8 (33.3**	**11 (45.8)**	**19 (79.2)**	**9 (37.5)**	**11 (45.8)**	**13 (54.2)**	**12 (50)**	

STarC, Strategic Readiness Center Course; ATTC, Army Trauma Training Center; NTTC, Navy Trauma Training Center; ERSS, Expeditionary Resuscitative Surgical System; USMC R2, United States Marine Corps Role 2; S2T2, Shipboard Surgical Trauma Training; GST, Ground Surgical Team; NATO SOST, North Atlantic Treaty Organization Special Operations Surgical Team; SME, Subject Matter Expert; AAR, After Action Report; TCCC, Tactical Combat Casualty Care; DCR, Damage Control Resuscitation; DCS, Damage Control Surgery.*Number of training days non-standardized and depends on the field exercise and USMC command. Usuallly between two and five days.

A gap commonly identified was that the current available courses focus on the GWOT and Counter Insurgency operations requirements of the last two decades, where short casualty holding times for surgical teams and rapid aeromedical evacuation evolved to be a standard. These standards will not exist in the future operating environment as has been witnessed in the Russo-Ukrainian War.^23–31^

Another major gap identified was a lack of preparing teams for potential future Large Scale Combat Operations (LSCO). LSCO is a diverse problem set and likely to be characterized by limited opportunities for rapid aeromedical evacuation. This analysis also identified gaps in medical service curricula addressing R2/ARSC surgical team’s ability to provide definitive surgical care (0% of courses), austere critical care (25%), and prolonged holding (0%). There was also a lack of standardized procedural skills training for non-surgeon ARSC team members, with only 50% including this curricular element. Five (62.5%) of eight courses simulated the operational environment for surgical teams, three (37.5%) included familiarization with night operations, and two (25%) simulated tactical exposure in the training. Other major gaps were the lack of critical skills cross training, intraoperative surgical assist training for non-surgeons, and curricular elements incorporating ethical considerations and the nonmedical challenges of the ARSC environment.

Through an iterative process of 24 meetings over 14 months, the ARSC WG identified the best practices needed to deploy successfully in austere environments. Ten modules comprised 20 TLOs and 259 ELOs were approved by the CoSCCC in February 2024.^26^ The final Joint ARSC Curriculum was published in July 2024^32^; the final product is available here (JTS_ARSC_Learning Objectives.pdf) and online at https://deployedmedicine.allogy.net/learner/collections/featured/337246fc-fc70-488e-8304-4ed47e507449/contents/3996

## DISCUSSION

The lack of a Joint (Army, Navy, Air Force, and Marine Corps) R2 surgical team training for the ARSC mission risks high-quality clinical care for wartime wounded and also poses a risk to the survival of surgical teams that deploy to these highly kinetic environments. Data from the Russo-Ukrainian War underscores the high-risk nature of these environments and the ongoing, evolving threat to medical teams.^27^^,^^29–31^ Through a comprehensive iterative Joint process led by the JTS with highly qualified SMEs, a Joint Force R2/ARSC curriculum was developed. Major curricular gaps identified in current service courses included nonstandard and inconsistent procedural skills taught to non-surgeon caregivers, inconsistent operational and tactical training, and limited or no curricular elements designed to prepare teams for the potential of LSCO such as prolonged holding, critical care, and definitive surgery in austere environments. In short, existing courses are primarily preparing ARSC and R2 surgical teams for the last war. Not anticipating the complexities of future wartime environments and combat casualty care during LSCO is a shortfall of current training paradigms.

In addition to relevant experience in trauma care, surgeons and other providers who have deployed to R2 forward surgical environments have been the biggest advocates for the development and wide implementation of standardized R2 team training.^6^^,^^9^^,^^14^^,^^15^^,^^33^ There are many reasons for this. The heuristics that forward-deployed caregivers develop when caring for patients in standard hospital environments result in cognitive biases that are amplified when deployed and risk errors in patient care. Each of the authors have approximately 10-20+ years each of training military caregivers for austere environments. In our experience, training in simulated operational and resource limited environments puts all team members in an “austere mindset” and exposes teams to these cognitive biases, improving both individual and team-based critical thinking skills. Team training also improves team dynamics by facilitating the development open and effective communication, and builds trust, empowerment, and accountability within the team.^33^ Finally, team training teaches the R2/ARSC team to communicate with the commanders of the operational units they support, facilitating both integration and expectation management of their capabilities.^9^

The irony of the lack of standardized ARSC training is that the services recognize the importance of these small surgical teams given the fact that so many have been developed and deployed during CENTCOM, AFRICOM, and INDOPACOM operations. The lack of a joint training program that fully prepares these teams for the strategic, operational, and tactical challenges of far forward, austere, or resource limited environments could have resulted from a failure of communication, or a lack of mission understanding among medical leaders. Because of the complexities of the Military Health System (MHS) with both Garrison and Operational missions, R2-forward surgical/ARSC teams are occasionally left orphaned of leadership. For example, a surgeon assigned to one of these teams could work in a DHA hospital, get assigned to a R2-forward surgical team that is owned by U.S. Army Forces Command, then deploy into a Combatant Command and get aligned under a medical brigade; there is a chance that the surgeon meets their team for the first time when they arrive in the theater of operations. This would hardly be considered “best practice” by medical or nonmedical leaders alike. This scenario is not uncommon—the authors know of instances that occurred during the drafting of this manuscript where a surgeon deployed with a team in theatre without ever having undergone the required platform team training as an individual or with the team with whom they deployed. However, the potential negative impact of changing personnel in theatre is lessened if R2/ARSC team training is standardized across the Joint Force. A continued irony in military medicine is that medical units can exist in paradox to the common military mantra of “train like you fight” because patient care typically occurs in standard military hospitals, not in austere environments. Whereas warfighters and their commanders are overall laser-focused on mission training in austere environments similar to the deployed environment. However, as a result of over 10 years of JTS and military service SME advocacy for Joint R2/ARSC mission training,^14^^,^^15^ and the ARSC WG curriculum presented here, the JTS will host the first five Joint R2/ARSC 12-day training courses in 2026, in San Antonio, TX. The JTS Joint Expeditionary Trauma Training (JETT) Course, will incorporate the best practice curriculuar elements described here, mitigating risk to the Joint Force; risk to casualty care, risk to the military mission, and risk to the surgical team.

Given current global threats, it is increasingly imperative that military medicine improve training, education, and resourcing for forward surgical and resuscitative care. In potential future operating environments, the lack of air superiority will limit re-supply and aeromedical evacuation; capabilities that were routine during the last 20 years of war. High casualty volumes of LSCO will challenge the logistics of patient evacuation and critical resupply. Therefore, any far-forward R2 surgical team on a future LSCO battlefield will be tasked to provide DCR, DCS, critical care, and definitive surgical capabilities with limited resources for prolonged periods of time.^24–27^ It must be a military medicine imperative to train forward surgical teams for these challenges.

Ideally, ARSC teams should be “expert teams of individual experts” which combine their talents and skills into a collective whole to best provide for patients.^34^ The use of small, mobile surgical assets by U.S. medical forces puts surgical capability closer to injured service members and decreases the time of evacuation from the POI to the operating table. However, splitting these teams into smaller, more agile elements compared with the surgical assets found in conventional R2 and R3 medical units comes with a significant decrease in surgical scope, capability, and patient holding capacity.^6^

Recent conflicts demonstrate that the U.S. military has geographic operations that are widely dispersed, encouraging the planning and fielding of small surgical teams. This trend is likely to continue on the future battlefield characterized by a range of low intensity conflict to high intensity LSCO. As such, small surgical teams will need to be more agile than ever. It might seem counterintuitive that an austere surgical team could have such an impact during LSCO if casualties arrive by the hundreds. Counterintuitively, it is precisely their light footprint, mobility, and agility that may make them ideal to adapt to this potential future operating environment. As seen in the Ukraine war, medical capabilities are likely to be targeted, therefore multiple highly mobile teams are used close to the “forward line of battle,” highlighting the importance R2/ARSC tactical exposure. Furthermore, during GWOT, ARSC teams routinely augmented static larger R2 military treatment facilities to respond to mass casualty (MASCAL) situations, as exemplified by the documented MASCAL events on the USS Bataan (2017)^35^ and at the Hamid Karzai International Airport (HKIA) in Kabul during the 2021 Afghanistan withdrawal.^36^ In both these instances several single surgeon ARSC teams worked together and sometimes interchangeably, to provide a unified MASCAL response. At HKIA, six surgical teams, most of which had never trained together, rapidly aggregated to respond to a terrorist attack generated MASCAL event where 63 casualties were treated, 15 emergency operations were performed, and over 100 units of blood products were transfused.^36^

Traditionally, preparation for deployment is accomplished through the maintenance of clinical skills at MTFs, often coupled with pre-deployment just-in-time clinical experiences where individual team members, or sometimes entire teams, are rotated through U.S.-based trauma centers to expose forward-deployed caregivers to patients with traumatic injuries.^14^^,^^15^^,^^37^^,^^38^ However, to prepare R2 forward surgical/ARSC teams for the anticipated challenges of LSCO, *clinical skills alone are insufficient for the effective deployment of small, minimally resourced, highly mobile surgical teams.*^6^^,^^39^ This point cannot be overstated.

The Joint R2/ARSC curriculum developed by the ARSC WG and the forthcoming JETT Course promote a learner-centric design that utilizes blended learning approaches and technology, increases access and usability of educational resources, and enables self-directed continuous learning to minimize knowledge and skills decay. The modules and learning objectives approved by the working group and CoSCCC^26^ are minimum standards designed to be implemented in different ways by the service-level courses. They are organized in the manner presented here to ensure completeness. However, a single surgical trauma scenario could incorporate numerous learning objectives from various modules and not necessarily follow the sequence laid out in the approved list. The intent is that this curriculum helps curriculum developers, course directors and teams effectively tailor and conduct clinically and operationally relevant, pre-deployment and readiness training for ARSC and R2 surgical teams.

**Figure 2.**
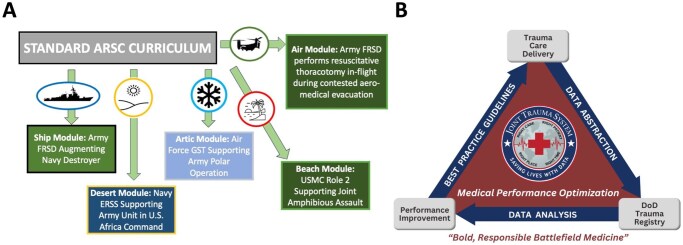
(A) Examples of joint course modules. (B) Joint trauma system operational cycle.

The curriculum described herein is only the first step to creating a Joint R2/ARSC course. Regardless of service R2 capability, the clinical care provided by R2/ARSC teams is not inherently different and based on JTS CPGs. What can be different is the environment in which resuscitative surgical care is performed. For example, a critical component of the Navy ERSS course is a scenario where surgical care is provided on a small Role 1 capable naval warship.^33^ An Air Force team might have a scenario where life-saving surgical care is provided in-flight.^40^ Other potential environments, depending on the mission, include buildings of opportunity, hasty field conditions, as well as arctic (cold), desert (dry), jungle (humid), and beach environments. The ideal joint R2/ARSC course would have modules simulating each of these environments to tailor the training to the mission or environment (**Figure 2A**). The WG also felt that critical components of any R2/ARSC courses is that the course must be (1) facilitated by experienced SMEs with deployment experience to ensure course quality control, and (2) after each simulated mission or scenario a SME led structured after-action review is performed where the teams learn how to analyze “the mission” to identify their strengths, weaknesses, and opportunities for improvement as a team to learn from their mistakes, and reinforce what they did well to continually improve individual and team performance.

Importantly, the joint curriculum incorporates R2/ARSC training into the JTS Operational Cycle (**Figure 2B**) of *Trauma Care Delivery* with documentation that gets abstracted into the *Department of Defense Trauma Registry* (DoDTR); from data analysis of the information that is in the DoDTR, *Performance Improvement* or *Medical Performance Optimization* occurs through CPGs, training, and education to save lives and improve trauma care delivery on the battlefield.^41^ Not only does this joint curriculum incorporate JTS CPG care standards that have saved lives on the battlefield,^42^^,^^43^ but the best practices of current available courses are also included. Furthermore, it also includes curricular elements to prepare ARSC and R2 surgical teams for the anticipated challenges of LSCO. Finally, the curriculum is designed to enhance confidence and trust-building among all ARSC adept personnel across military medicine, consistent with the goal of increased interoperability. ­Ultimately, the goal of the JTS is to further incorporate these Joint curriculum standards into the JTS Operational Cycle (**Figure 2B**) including

1. demonstrate, evaluate, endorse, & train ARSC/Role 2 curricula and courses;
2. rapidly adapt pre-deployment training courses based on contemporary DoDTR data, CPG updates, and JTS process improvement initiatives; and
3. promulgate best practices from other service’s ARSC/R2 curricula and courses.

Starting in 2026, the JTS will begin routinely demonstrating and teaching the JETT Course for the Joint Force. Through the process of reviewing and endorsing the Service’s current courses, the JTS could also help rapidly promulgate curriculum updates to the JETT Course and Service courses based on DoDTR data informed process improvement initiatives and best practices identified from the Services.

A potential limitation of the curriculum development process described here is that Individual unit ARSC/R2 training courses unknown to the WG could have been not included in the curricular best practice/gap analysis. Although possible, this is unlikely given the multidisciplinary make-up and the combined experience of the WG SMEs and the extensive analysis that was completed on existing courses. Another limitation is the inability to compare clinical outcomes from different pre-deployment training courses, or lack of pre-deployment training. That said, this curriculum sets a minimum standard for service ARSC team training, something that does not exist at present.

This Joint curriculum helps ensure interoperability in education, training, outfitting and operational utilization, while continuing to allow for service- and mission-specific customization as required. A joint minimum standard is long overdue. The forthcoming JETT Course will be essential to meet the gaps in training that have been validated within the DoW and independently through external reviews of the current battlefield medical support system.

## CONCLUSION

Military medicine lacks a joint standard for training ARSC and R2 surgical teams which is a risk to force and risk to mission. Existing service courses, if taken, are preparing current ARSC/R2 teams for the war already fought—not the future fight. Through a comprehensive iterative SME-led process, the JTS developed the first Joint R2 forward surgical/ARSC curriculum which incorporates current best practices and addresses significant training gaps in preparing Joint Force surgical teams for LSCO. As a result of this project and other advocacy efforts, the first Joint R2/ARSC team training will be realized in the forthcoming JTS JETT Course.

## Supplementary Material

usaf576_Supplementary_Data

## Data Availability

The data that support the findings of this study are available on request from the corresponding author.

## INSTITUTIONAL CLEARANCE

Institutional approval granted by DHA Public Affairs Office.

## PRIOR PRESENTATION

This study was originally presented as a poster at the Military Health System Research Symposium in Kissimmee, Florida, August 2024.

## References

[1] Wren SM , Wild HB, Gurney J, et al A consensus framework for the humanitarian surgical response to armed conflict in 21st century warfare. JAMA Surg. 2020;155:114–21. 10.1001/jamasurg.2019.4547 31722004 PMC6865259

[2] World Health Organization Special Situation Report: Mosul Crisis, Iraq. Issue No 26: 9–23 July 2017. Accessed March 4, 2025. https://www.emro.who.int/images/stories/who_special_situation_report_on_mosul_crisis_09_to_23_july_2017_cleared.pdf? ua=1.

[3] Joint Chiefs of Staff. Joint Publication 4-02 Joint Health Services. Joint Forces Development; 2018; 28 December.

[4] Joint Chiefs of Staff. Joint Publication 4-02 Joint Health Services. Joint Forces Development; 2023, 29 August.

[5] Cubano MA. Emergency War Surgery, 5th US Revision: Government Printing Office; 2018.

[6] Gurney JM , Jensen SD, Gavitt BJ, et al Committee on surgical combat casualty care position statement on the use of single surgeon teams and invited commentaries. J Trauma Acute Care Surg. 2022;93:S6–S11. 10.1097/ta.0000000000003676 35522930

[7] Tadlock MD , Mosely DS, Baker JB, Edson TD, Gurney JM. The development of military medicine’s FIRST comprehensive joint austere resuscitative surgical care curriculum for role 2 surgical teams. Mil Med. 2025;190:e1336–e9. 10.1093/milmed/usaf074 40173030

[8] Baker JB , Northern DM, Frament C, et al Austere resuscitative and surgical care in support of forward military operations—joint trauma system position paper. Mil Med. 2021;186:12–7. 10.1093/milmed/usaa358 33185671

[9] Bingham J , Satterly S, Eckert M. Austere resuscitative and surgical care in modern combat operations. Curr Trauma Rep. 2021;7:53–9. 10.1007/s40719-021-00214-0

[10] Austere Resuscitative and Surgical Care (ARSC) Clinical Practice Guideline. October 30, 2019. Available at: https://jts.health.mil/index.cfm/PI_CPGs/cpgs. Accessed January 14, 2025.

[11] Kotwal RS , Howard JT, Orman JA, et al The effect of a golden hour policy on the morbidity and mortality of combat casualties. JAMA Surg. 2016;151:15–24. 10.1001/jamasurg.2015.3104 26422778

[12] Shackelford SA , Del Junco DJ, Mazuchowski EL, et al The golden hour of casualty care: rapid handoff to surgical team is associated with improved survival in war-injured US service members. Ann Surg. 2024;279:1–10. 10.1097/sla.0000000000005787 36728667

[13] Vasquez M , Edson TD, Lucas DJ, Hall AB, Tadlock MD. The impact of the Maritime deployment cycle on the surgeon’s knowledge, skills, and abilities. Mil Med. 2023;188:e1382–e8. 10.1093/milmed/usac316 36260423

[14] Tyler JA , Ritchie JD, Leas ML, et al Combat readiness for the modern military surgeon: data from a decade of combat operations. J Trauma Acute Care Surg. 2012;73:S64–S70. 10.1097/TA.0b013e3182625ebb 22847097

[15] Schwab CW. Winds of war: enhancing civilian and military partnerships to assure readiness: white paper. J Am Coll Surg. 2015;221:235–54. 10.1016/j.jamcollsurg.2015.04.014 26206632

[16] D Berwick , A Downey, E Cornett, editors. *A National Trauma Care System: Integrating Military and Civilian Trauma Systems to Achieve Zero Preventable Deaths after Injury*. 2016. The National Acadamies Press, Washington D.C 27748086

[17] Joint Requirements Oversight Council Memorandum (JROCM) 125-17, “Forward Resuscitative Care in Support of Dispersed Operations DOTmLPF-P Change Recommendation,” dated December 11, 2017.

[18] Inspector General (IG) Audit “Audit of Training of Mobile Medical Teams in the U.S. Indo-Pacific Command and U.S. Africa Command Areas of Responsibility” (Report No. DODIG-2020-087) dated June 8, 2020. Available at: https://www.dodig.mil/reports.html/Article/2213982/audit-of-training-of-mobile-medical-teams-in-the-us-indo-pacific-command-and-us/. Accessed January 1, 2024.

[19] Section 708 of the National Defense Authorization Act for Fiscal Year 2017 (Public Law 114-328): “Establishment of Joint Trauma Education and Training Directorate” Interim Report and Implementation Plan. February 14, 2018. Available at: https://www.health.mil/Reference-Center/Reports/2018/02/14/Joint-Trauma-Education-and-Training-Directorate#:∼:text=This%20interim%20report%20and%20implementation,(d)%20Personnel%20Management%20Plan. Accessed January 2, 2024.

[20] Committee on Surgical Combat Casualty Care Meeting Minutes. https://jts.health.mil/index.cfm/committees/cosccc/mtg_minutes. Accessed May 2022 through May 2024.

[21] Course Information, Emergency War Surgery Course. https://www.health.mil/Military-Health-Topics/Education-and-Training/DMRTI/Course-Information/Emergency-War-Surgery-Course. Accessed January 2nd, 2025.

[22] Andreatta P , Bowyer MW, Ritter EM, Remick K, Knudson MM, Elster EA. Evidence-based surgical competency outcomes from the clinical readiness program. Ann Surg. 2023;277:e992–e9. 10.1097/SLA.0000000000005324 34879053

[23] Remondelli MH , Remick KN, Shackelford SA, et al Casualty care implications of large-scale combat operations. J Trauma Acute Care Surg. 2023;95:S180–S184. 10.1097/ta.0000000000004063 37420334 PMC10389308

[24] Meeting Minutes, Committee on Surgical Combat Casualty Care. March 22-23, 2023. San Antonio Texas. https://jts.health.mil/index.cfm/committees/cosccc/mtg_minutes. Accessed December 13, 2024.

[25] Meeting Minutes, Committee on Surgical Combat Casualty Care. August 24-25, 2023. San Antonio Texas. Available at: https://jts.health.mil/index.cfm/committees/cosccc/mtg_minutes. Accessed December 13, 2024.

[26] Meeting Minutes, Committee on Surgical Combat Casualty Care. March 27–28, 2024. San Antonio Texas. Available at: https://jts.health.mil/index.cfm/committees/cosccc/mtg_minutes. Accessed December 13, 2024.

[27] Meeting Minutes, Committee on Surgical Combat Casualty Care. November 6–7, 2024. San Antonio Texas. Available at: https://jts.health.mil/index.cfm/committees/cosccc/mtg_minutes. Accessed December 13, 2024.

[28] Powell EK , Betzold R, Hardin RD, et al The incidence, outcome, and treatment of advanced organ failure and support after trauma: a review with implications for future large-scale combat operations. J Trauma Acute Care Surg. 2025;99:S133–S142. 10.1097/ta.0000000000004685 40539974

[29] Bongartz T. “Medical Realities of Large-Scale Combat Operations: The Ukraine Experience, Interim Report of the Pathfinder Working Group Committee on Surgical Combat Casualty Care., San Antonio, TX, November 7–8, 2024.

[30] Polk TM. “Defining the Problem of Combat Casualty Care in the Drone Era: lessons from Ukraine Committee on Surgical Combat Casualty Care., San Antonio, TX, 13–14 May 2025.

[31] Meeting Highlights, Committee on Surgical Combat Casualty Care. May 13-14, 2025. Available at: https://jts.health.mil/assets/docs/cosccc/CoSCCC_Highlights_FINAL_24_July_2025.pdf. Accessed August 15, 2025.

[32] Austere Resuscitative Surgical Care Learning Objectives. July 22, 2024. Available at: https://deployedmedicine.allogy.net/learner/collections/featured/337246fc-fc70-488e-8304-4ed47e507449/contents/3996.Accessed January 14th, 2025.

[33] Vicente DA , Ugochukwu O, Johnston MG, Craft C, Damin V, Tadlock MD. Preparing austere Maritime surgical teams for deployment during the COVID-19 global pandemic: is it time to change the TrainingPipeline? Mil Med. 2021;186:e873–e878. e873-e 10.1093/milmed/usaa574 33399864 PMC7928804

[34] Andreatta PB , Graybill JC, Renninger CH, Armstrong RK, Bowyer MW, Gurney JM. Five influential factors for clinical team performance in urgent, emergency care contexts. Mil Med. 2023;188:e2480–e8. 10.1093/milmed/usac269

[35] Miller BT , Lin AH, Clark SC, Cap AP, Dubose JJ. Red tides: mass casualty and whole blood at sea. J Trauma Acute Care Surg. 2018;85:S134–S139. 10.1097/TA.0000000000001831 29443862

[36] Bozzay Joseph D , Murphy Timothy P, Baird Michael D, et al The last days: the medical response of United States and allied military teams during the Afghanistan exodus. J Trauma Acute Care Surg., 2023;95:S13–S18. 10.1097/ta.0000000000004062 37246291 PMC10389402

[37] Tadlock MD , Carr M, Diaz J, et al How to maintain the readiness of forward deployed caregivers. J Trauma Acute Care Surg. 2021;90:e87–e94. 10.1097/ta.0000000000003054 33405471

[38] Lee JJ , Hall AB, Carr MJ, MacDonald AG, Edson TD, Tadlock MD. Integrated military and civilian partnerships are necessary for effective trauma-related training and skills sustainment during the inter-war period. J Trauma Acute Care Surg. 2022;92:e57–e76. 10.1097/TA.0000000000003477 34797811

[39] Lesperance RN , Adamson S, Gurney JM. Lessons learned during prolonged care of combat casualties by a minimally manned surgical team. Mil Med. 2023;188. 10.1093/milmed/usab299 34272956

[40] DuBose JJ , Stinner DJ, Baudek A, et al Life and limb In-Flight surgical intervention: fifteen years of experience by joint medical augmentation unit surgical resuscitation teams. J Spec Oper Med. 2020;20:47–52. 10.55460/SI6S-XHCZ 33320312

[41] Gurney JM , Tadlock MD, Van Gent J-M. Joint trauma system: behind the scenes. Mil Med. 2025; Feb 27190:e464–e466. 10.1093/milmed/usaf014 39937622

[42] Eastridge BJ , Costanzo G, Jenkins D, et al Impact of joint theater trauma system initiatives on battlefield injury outcomes. Am J Surg. 2009;198:852–7. 10.1016/j.amjsurg.2009.04.029 19969141

[43] Eastridge Brian J , Wade Charles E, Spott Mary A, et al Utilizing a trauma systems approach to benchmark and improve combat casualty care. J Trauma. 2010;69:S5-9. 10.1097/TA.0b013e3181e421f3 20622620

