# Supplementary material for: Fighting to Train—Implementation of a Train Like You Fight Joint Role 2 Austere Surgical Care Curriculum
Source: Mil Med. 2025 Dec 4;191(7-8):e1536–45. doi: 10.1093/milmed/usaf576 (PMC13331495; doi:10.1093/milmed/usaf576)
Supplement: usaf576_Supplementary_Data [file usaf576_supplementary_data.zip › usaf576_Supplementary_Data/JTS_ARSC_Learning Objectives.pdf]

ARSC

AUSTERE RESUSCITATIVE SURGICAL CARE

LEARNING OBJECTIVES

22 JULY 2024

Committee on Surgical Combat Casualty Care (CoSCCC)

Austere Resuscitative Surgical Care Provided by Deployed Surgical Teams

Austere Resuscitative Surgical Care (ARSC) is an advanced medical & surgical capability delivered by small surgical teams with limited resources. ARSC bridges the gap between injury and definitive surgical care thus decreasing battlefield mortality. ARSC teams support military operations by decreasing risk to force.

> ***"Good surgery must be done as far forward as possible. If it is too good, in the sense of too elaborately equipped, it will not be far enough forward, and if it is too far forward it will not be good enough."***
>
> **William H. Ogilvie, 1887-1971**

Lifesaving resuscitation and surgical care in the forward deployed, austere, resourced limited environment provided by teams ranging from 6 to 20 personnel is a capability bundle referred to as: Austere Resuscitative Surgical Care (ARSC). There are many teams in each of the Services that provide this capability; however, there have been no joint standards for training these teams.

ARSC teams bridge the battlefield Roles of Care and are an essential part of the deployed military trauma system. ARSC teams most commonly function as Role 2 surgical teams bridging the gap between point of injury (POI) care and more definitive surgical care that occurs at Role 3 Military Treatment Facilities (MTFs). ARSC teams fall within the spectrum of Role 2 forward resuscitative care but may be less resourced and further forward than a fully manned and equipped Role 2. Each Service has a variety of names/capabilities for these forward surgical teams which has created some confusion as many teams with different names deploy in support of Geographic Combatant Commands. The intent of using the ARSC term is to standardize the basics of this capability as well as the training and skills required to improve survival from battlefield injury.

As mentioned above, ARSC is an overarching term used to describe the primary mission of these forward deployed teams. ARSC capabilities are more agile and maneuverable than standard Role 2s, allowing them to provide a surgical/resuscitative capability closer to POI in battlefield/contingency environments. ARSC teams are assets to military commanders because they decrease the risk to force by improving survival from injury and other military health threats. However, ARSC/Role 2 surgical capabilities, if not manned, trained and equipped appropriately, may introduce risk to force and risk to mission if commanders assume there is a capability in their AOR that does not have the skills these teams need.[1]

Data from the Joint Trauma System demonstrates that over 24,000 surgical procedures were performed at Role 2 MTFs (by ARSC teams) from 2003 to 2018 in Afghanistan and Iraq. ARSC teams provide lifesaving capabilities that include, but are not limited to, damage control resuscitation, damage control surgery, definitive surgery, massive transfusion, transfusion of

[1] https://jts.health.mil/assets/docs/cosccc/CoSCCC_Position_Statement_on_Single_Surgeon_Teams.pdf

fresh whole blood from Walking Blood Banks, management of orthopedic injuries, treatment of communicable diseases, and management of other military health threats.

The Services recognize the importance of the ARSC/Role 2 surgical capabilities and thus have created multiple constructs of ARSC teams over the past 20 years. Despite the frequent deployment of these teams, there remains no joint training program that fully prepares these teams for the strategic, operational, and tactical challenges of the far forward, austere, or resource limited environments. This lack of training standards has been identified by the Inspector General (IG), Report No. DODIG-2020-087 “Audit of Training of Mobile Medical Teams in the U.S. Indo-Pacific Command and U.S. Africa Command Areas of Responsibility” June 8, 2020, and results in current and future unacceptable risks to casualties cared for by these teams. Setting the conditions for R2 team success includes operational considerations such as communication, team cohesion, and leadership. Surgical expertise, high quality resuscitation, and team cohesion are foundational for excellent clinical care at Role 2 MTFs in austere, resource limited, or expeditionary environments.

This Training Supplement was created to address the lack of a Joint Role 2 Austere Surgical Team Training Course that focuses on clinical (surgical and resuscitative) care in a challenging tactical environment. It provides the minimum standards established by the ARSC Working Group (ARSC WG) and approved by the Committee on Surgical Combat Casualty Care (CoSCCC) in the form of Terminal Learning Objectives (TLOs) and Enabling Learning Objectives (ELOs) to help curriculum developers, course directors and teams effectively conduct clinically and operationally relevant, pre-deployment and readiness training for ARSC/R2 surgical teams.[2] The course curriculum that emerged from the efforts of the ARSC WG is the Joint Expeditionary Trauma Training Course (JETT).[3] Questions on this training supplement or the JETT course can be directed to the Joint Trauma Education and Training Branch, Joint Trauma System at dha.jbsa.healthcare-ops.list.jts-jtet@health.mil.

The ARSC WG consisted of nominated representatives from the MILDEPs, FORSCOM and OJSS as voting members and dozens of other passionate and committed subject matter experts from both the DoD and civilian medical sectors. <u>We would like to formally thank all the members of the ARSC WG who committed hundreds of volunteer hours over two years to develop these ELOs, TLOs and the baseline curriculum</u>. The JTS looks forward to implementing this course when the authorities and funding streams for execution have been determined.

[2] INTENT & AUTHORITIES: The Joint Trauma System (JTS) formally chartered the Austere Surgical Care Working Group (ARSC WG) in 2022 in response to the dissolution of a 3-week course that met the clinical and operational objectives R2/ARSC environment. There was, and continues to be, a lack of consensus of who owns this validated requirement with regards to funding and execution. The authority for the Joint Trauma Education and Training (JTET) Branch of the JTS to develop a standardized combat casualty care instruction was codified in the NDAA 2017, Section 708 (b).

[3] The ARSC WG developed these learning objectives and a curriculum IAW standards directed within the Department of Defense Instruction (DoDI) 1322.24, “Medical Readiness Training (MRT),” dated February 15, 2022, and Joint Requirements Oversight Council Memorandum (JROCM) 125-17, “Forward Resuscitative Care in Support of Dispersed Operations DOTmLPF-P Change Recommendation,” dated December 11, 2017, Action 7.

Module Descriptions

#	MODULE TITLE	MODULE DESCRIPTION
01	**Team Development and Dynamics**	Actions that enable the group to function optimally as a team and integrate various skillsets into a cohesive element to manage complex surgical patients in an austere setting.
02	**Trauma System Integration**	Actions that ensure the ARSC team understands how the team fits into the overall Combatant Command Trauma System.
03	**Clinical Operations in the Austere Environment**	Tactical actions that are essential for effective clinical operations.
04	**Preparing to Receive Patients**	Actions which are integral to preparation for effective and efficient management of patient care and flow.
05	**Patient Receiving and Management**	Actions taken individually and as a team upon receiving casualties.
06	**Surgical Resuscitation in the Austere Environment**	Actions and knowledge required for successful surgical operations in the austere environment.
07	**Post-surgical Care**	Actions and knowledge required for management of post-operative patients in the austere environment.
08	**Austere Critical Care**	Actions required to address management of both surgical and non-surgical critically ill patients in the austere environment.
09	**Patient Packaging and Transport**	Actions that are required to prepare a patient for evacuation.
10	**Prolonged Patient Holding**	Actions that are necessary when patient evacuation is delayed and patients require unanticipated care in an austere setting.

LEARNING OBJECTIVES

Module 1 – Team Development and Dynamics

TLO	#	ELO
Given an austere resuscitative surgical care (ARSC) scenario, perform team development and dynamics in accordance with Defense Committees on Trauma (DCoT), Committee on Surgical Combat Casualty Care (CoSCCC) and Joint Trauma System (JTS) Guidelines	1	Analyze the relationship between ARSC leadership structure and team dynamics
	2	Demonstrate small unit communication and coordination
	3	Differentiate between key indicators of clinical proficiency for all ARSC team members
	4	Analyze relevant CPGs as it relates to team members' skills and practice
	5	Perform team-level cross training in critical skills
	6	Describe elements of non-traditional roles for team members in the austere environment
	7	Demonstrate crew resource management (CRM)
	8	Demonstrate incorporation of supporting personnel
	9	Explain the importance of ARSC team members familiarization with the military decision-making process and its impact on operational planning
	10	Distinguish between the application of ethical principles in clinical practice and combat casualty care
	11	Discuss key components of individual and ARSC team resilience
	12	Understand team members' scope of practice (anesthesia, nursing, medics/corpsmen, etc.) and be able to coordinate with team members to optimize surgical care and medical treatment. For example, respond to request to assist intubation, set up IV pumps, initiate WBB, etc.

LEARNING OBJECTIVES

Module 2 – Trauma System Integration

TLO		ELO
Given an austere resuscitative surgical care (ARSC) scenario, perform trauma system integration in accordance with Defense Committees on Trauma (DCoT), Committee on Surgical Combat Casualty Care (CoSCCC) and Joint Trauma System (JTS) Guidelines	1	Describe the deployed trauma system
	2	Describe relationship between unit location and continuum of care
	3	Describe local medical asset integration
	4	Describe the partner nation trauma system
	5	Demonstrate documentation and reporting
	6	Demonstrate integration of patient tracking system that includes patient identification and confidentiality
	7	Conduct Performance Improvement (PI)

LEARNING OBJECTIVES

Module 3 – Clinical Operations in the Austere Environment

TLO	ELO	
Given an austere resuscitative surgical care (ARSC) scenario, perform Clinical Operations in the austere environment in accordance with Defense Committees on Trauma (DCoT), Committee on Surgical Combat Casualty Care (CoSCCC) and Joint Trauma System (JTS) Guidelines	1	**Plan a CONOP by demonstrating mission analysis and COA development upon mission receipt**
	2	**Describe the team security posture**
	3	**Describe environmental factors that impact an ARSC team in an austere environment**
	4	**Plan for the effect of contingency/split operations on team capability and capacity**
	5	**Describe unit-specific tactical proficiency**
	6	**Develop a comprehensive logistics plan**
	7	**Demonstrate Team equipment familiarization and operation**
	8	**Describe medical equipment maintenance plan**
	9	**Describe non-medical equipment maintenance and power management**
	10	**Demonstrate a blood management operation**
	11	**Develop a narcotics management plan**
	12	**Describe accountability of sensitive items**
	13	**Develop and execute a comprehensive communications plan**
	14	**Describe the clinical decision-making process in an austere environment**
	15	**Develop and execute a military brief to Command authority**

LEARNING OBJECTIVES

Module 4 – Preparing to Receive Patients

TLO	ELO	
Given an austere resuscitative surgical care (ARSC) scenario, perform preparation for receiving patients in the austere environment in accordance with Defense Committees on Trauma (DCoT), Committee on Surgical Combat Casualty Care (CoSCCC) and Joint Trauma System (JTS) Guidelines	1	Conduct mission analysis to provide health service support
	2	Describe, communicate, and manage Command expectations (Commander SITREP)
	3	Describe medical rules of eligibility (MEDROE) planning for special patient populations
	4	Describe individual patient management (non-clinical)
	5	Describe structured team feedback process IAW Service-specific standard process
	6	Develop a patient flow plan
	7	Develop a MASCAL plan
	8	Develop a patient holding plan
	9	Develop a patient movement/evacuation plan
	10	Develop a communication plan
	11	Develop movement plan for patients to and from team location, utilizing non-team support personnel and assets
	12	Develop a CBRNE plan
	13	Develop a contingency plan
	14	Demonstrate equipment setup
	15	Demonstrate surgical rehearsal exercise prior to receiving patients (i.e., rehearsal of concept [ROC] drills)

Module 5 – Patient Receiving and Management

Module description –

TLO		ELO
**Given an austere resuscitative surgical care (ARSC) scenario, perform patient management in the austere environment in accordance with Defense Committees on Trauma (DCoT), Committee on Surgical Combat Casualty Care (CoSCCC) and Joint Trauma System (JTS) Guidelines**	1	**Demonstrate primary and secondary surveys with use of available diagnostic tools**
	2	**Perform patient flow during multiple casualty scenarios**
	3	**Perform triage of multiple complex surgical and non-surgical casualties**
	4	**Perform team communication during triage**
	5	**Execute MASCAL plan**
	6	**Distinguish between compressible and non-compressible hemorrhage**
	7	**Demonstrate damage control resuscitation (DCR)**
	8	**Perform REBOA placement**
	9	**Execute blood management program and walking blood bank**
	10	**Demonstrate management of airway emergencies**
	11	**Describe management of ocular emergencies**
	8	**Describe management of partial thickness burns**
	9	**Describe management of smoke inhalation**
	10	**Demonstrate management of soft tissue injuries**
	11	**Demonstrate management of lacerations**
	12	**Demonstrate management of contaminated soft tissue injuries**
	12	**Describe pain management**
	13	**Perform management of multiple simultaneous non-surgical patients**
	14	**Describe pitfalls when moving critical and/or post-operative patients in the austere environment**
	15	**Demonstrate patient movement drills**
	16	**Perform palliative care for expectant patients**
	17	**Demonstrate documentation of patient care**
	18	**Demonstrate structure team feedback process IAW Service-specific standard process**
	19	**Demonstrate management of logistics for non-surgical equipment and supplies**
	20	**Describe management of hazardous waste**

	21	Describe integration with mortuary affairs

TLO		ELO
Given an austere resuscitative surgical care (ARSC) scenario, perform management of special patient populations in the austere environment in accordance with Defense Committees on Trauma (DCoT), Committee on Surgical Combat Casualty Care (CoSCCC) and Joint Trauma System (JTS) Guidelines	1	Describe urgent/emergent care of host nation VIP patients
	2	Describe urgent/emergent care of high value targets (HVTs)
	3	Describe urgent/emergent care of Partner forces
	4	Describe urgent/emergent care of friendly forces non-military personnel
	5	Describe urgent/emergent care of detainees
	6	Describe urgent/emergent care of noncombants (or civilians)
	7	Demonstrate urgent/emergent care of pediatric patients
	8	Describe urgent/emergent care Ob/Gyn patients
	9	Demonstrate urgent/emergent care of Military Working Dogs (MWD)

TLO		ELO
Given an austere resuscitative surgical care (ARSC) scenario, perform management of non-surgical musculoskeletal injuries in the austere environment in accordance with Defense Committees on Trauma (DCoT), Committee on Surgical Combat Casualty Care (CoSCCC) and Joint Trauma System (JTS) Guidelines	1	Demonstrate urgent/emergent care of suspected pelvic injury
	2	Demonstrate urgent/emergent care of suspected spinal injury
	3	Identify signs of limb ischemia
	4	Identify signs and symptoms of compartment syndrome
	5	Demonstrate urgent/emergent care of open fractures
	6	Demonstrate urgent/emergent care of fractures
	7	Describe urgent/emergent care of shoulder dislocations
	8	Describe urgent/emergent care of hip dislocations
	9	Describe urgent/emergent care of knee dislocations
	10	Demonstrate wound packing of extremity wounds
	11	Demonstrate splint placement
	12	Demonstrate placement of lower extremity traction splint
	13	Demonstrate use of analgesia for musculoskeletal injuries
	14	Demonstrate use of local anesthesia for musculoskeletal injuries

	15	**Demonstrate use of axillary regional anesthesia for upper extremity injuries**
	16	**Demonstrate use of upper extremity regional anesthesia for hand injuries**
	17	**Demonstrate use of regional anesthesia for finger injuries**
	18	**Demonstrate use of femoral regional anesthesia for lower extremity injuries**
	19	**Demonstrate use of popliteal regional anesthesia for lower extremity injuries**
	20	**Conduct post intervention exam of any extremity injury**

TLO	ELO	
**Given an austere resuscitative surgical care (ARSC) scenario, perform management of urgent disease and non-battle injuries in the austere environment in accordance with Defense Committees on Trauma (DCoT), Committee on Surgical Combat Casualty Care (CoSCCC) and Joint Trauma System (JTS) Guidelines**	1	**Demonstrate use of theatre-specific, service-specific references and disease threat assessments for anticipated deployed environment**
	2	**Describe management of endemic infectious diseases based on medical intelligence planning**
	3	**Synthesize the environmental considerations for clinical management of all DNBI care**
	4	**Demonstrate initial treatment of infection**
	5	**Identify signs of sepsis**
	6	**Describe management of cold-related injuries**
	7	**Describe management of heat-related injuries**
	8	**Describe management of envenomation injuries (local specific)**
	9	**Describe management of neuropsychiatric conditions**
	10	**Describe management of dental conditions**

AUSTERE RESUSCITATIVE SURGICAL CARE

LEARNING OBJECTIVES

Module 6 – Surgical Resuscitation in the Austere Environment

TLO	ELO	
Given an austere resuscitative surgical care (ARSC) scenario, perform damage control surgical resuscitation in the austere environment in accordance with Defense Committees on Trauma (DCoT), Committee on Surgical Combat Casualty Care (CoSCCC) and Joint Trauma System (JTS) Guidelines	1	Perform management of logistics for surgical equipment and supplies
	2	Perform surgical decision-making process and surgical management of chest injuries
	3	Perform surgical decision-making process and surgical management of abdominal injuries
	4	Perform surgical decision-making process and surgical management of pelvic injuries
	5	Perform surgical decision-making process and surgical management of extremity injuries
	6	Perform surgical decision-making process with focus on the understanding of techniques and limitations of basic neurosurgery procedures in the ARSC environment
	7	Perform surgical decision-making process and surgical management of neck injuries
	8	Perform surgical decision-making process and surgical management of vascular injuries
	10	Describe the impact of spinal injuries on surgical resuscitation in the austere environment
	11	Perform temporary closure techniques of the abdomen and chest and describe alternate methods for surgical wound management
	12	Perform surgical management of burn casualties
	13	Perform anesthesia for surgical patients
	14	Demonstrate the ability to designate surgical assistance tasks to non-surgical team members
	15	Demonstrate the ability to delegate surgical resuscitation tasks to non-surgical team members
	16	Perform documentation of operative management
	17	Perform decision-making and actions for expectant patients
	18	Describe alterations in team dynamics and priorities of care when the team is given a no-notice movement order

TLO	ELO	
Given an austere resuscitative surgical care (ARSC) scenario, perform assistant training for non-surgeon team	1	Demonstrate surgical principles of surgical exposure
	2	Describe techniques for illumination of operative field
	3	Describe surgical maneuvers of tension/counter-tension
	4	Demonstrate basic maneuvers of surgical dissection

members in the austere environment in accordance with Defense Committees on Trauma (DCoT), Committee on Surgical Combat Casualty Care (CoSCCC) and Joint Trauma System (JTS) Guidelines	5	Perform *surgical battle drills* with the team surgeon (i.e., clamp-clamp-cut-tie-tie-cut, stitch-cut, loading pledgets, etc.)
	6	Identify common instruments and equipment used by surgeons on the team
	7	Describe contents of team-based surgical load out
	8	Describe cross-training of anesthesia support skills for entire team, specifically the maintenance and troubleshooting of blood cooler and the use of an alternate storage method in the case of power failure
	9	Describe cross-training of anesthesia support skills for entire team, specifically the setup and priming of IV tubing
	10	Describe cross-training of anesthesia support skills for entire team, specifically methods for mixing Total IV Anesthesia (TIVA)
	11	Describe cross-training of anesthesia support skills for entire team, specifically the basics of surgical anesthesia in the event that there are multiple simultaneous surgical cases
	12	Describe cross-training of surgical nursing support skills for entire team
	13	Demonstrate alterations in team dynamics when a team member becomes a casualty

TLO	ELO	
Given an austere resuscitative surgical care (ARSC) scenario, perform surgical management of special patient populations in the austere environment in accordance with Defense Committees on Trauma (DCoT), Committee on Surgical Combat Casualty Care (CoSCCC) and Joint Trauma System (JTS) Guidelines	1	Demonstrate surgical decision-making process of Military Working Dogs (MWD)
	2	Demonstrate MARCH protocol for MWD
	3	Demonstrate use of ultrasound for MWD
	4	Describe basic anesthesia and analgesia for MWD
	5	Describe surgical decision-making process of pediatric patients
	6	Demonstrate MARCH protocol for pediatric patients
	7	Demonstrate use of ultrasound for pediatric patients
	8	Describe basic anesthesia and analgesia for pediatric patients
	9	Describe surgical decision-making process for Ob/Gyn patients
	10	Describe principles of humanitarian medicine and surgical care
	11	Describe final disposition effects of surgical decision-making process regarding enemy combatants, partner forces and noncombatants

LEARNING OBJECTIVES

Module 7 – Post-Surgical Care

TLO		ELO
Given an austere resuscitative surgical care (ARSC) scenario, perform post-surgical care in the austere environment in the austere environment in accordance with Defense Committees on Trauma (DCoT), Committee on Surgical Combat Casualty Care (CoSCCC) and Joint Trauma System (JTS) Guidelines	1	Perform field expedient reprocessing of surgical equipment IAW Wound Care CPG
	2	Perform reset of surgical table and supplies
	3	Demonstrate patient documentation
	4	Perform blood bank reconstitution
	5	Develop status brief to higher Command authority
	6	Describe infection control in the austere environment
	7	Describe decision-making process for repeat operations
	8	Demonstrate closed-loop communication with team on operative care and post-operative plan
	9	Demonstrate structured team feedback process of operative case IAW Service-specific standard process
	10	Describe hazardous waste disposal procedures
	11	Demonstrate implementation of maintenance plan for medical equipment

TLO		ELO
Given an austere resuscitative surgical care (ARSC) scenario, perform management of post operative complications in the austere environment in accordance with Defense Committees on Trauma (DCoT), Committee on Surgical Combat Casualty Care (CoSCCC) and Joint Trauma System (JTS) Guidelines	1	Describe signs, symptoms, and management of shunt/graft failure
	2	Describe signs, symptoms, and management of airway failure
	3	Describe signs, symptoms, and management infections requiring surgical intervention
	4	Describe signs, symptoms, and management of post operative bleeding
	5	Describe signs, symptoms, and management of abdominal compartment syndrome
	6	Describe signs, symptoms, and management of missed limb compartment syndrome after incomplete fasciotomy

Module 8 – Austere Critical Care

TLO	ELO	
Given an austere resuscitative surgical care (ARSC) scenario, perform austere critical care in the austere environment in accordance with Defense Committees on Trauma (DCoT), Committee on Surgical Combat Casualty Care (CoSCCC) and Joint Trauma System (JTS) Guidelines	1	Demonstrate monitoring of critically ill patients
	2	Describe hypothermia prevention and management of critically ill patients
	3	Demonstrate management of hypothermia patient
	4	Describe frostbite management of critically ill patients
	5	Describe ventilator management of critically ill patients to include invasive and non-invasive airway management
	6	Describe signs, symptoms, and management of sepsis
	7	Describe appropriate empiric antibiotic therapy IAW current JTS CPGs
	8	Describe signs, symptoms, and management of necrotizing soft tissue infections
	9	Describe goals and endpoints of resuscitation
	10	Describe methods for ongoing sedation, analgesia, and delirium IAW current JTS CPGs
	11	Describe management of fluid balance in critically ill patients
	12	Describe targeted resuscitation of burn patients IAW current JTS CPGs
	13	Describe continuous resuscitation of TBI patients IAW current JTS CPGs
	14	Identify expectant patients
	15	Describe process for managing expectant US patients
	16	Describe management of expectant non-US patients
	17	Perform palliative care for expectant patients
	18	Demonstrate enacting plan to appropriately care for body of at least one deceased patient, including Service members and non-US patients
	19	Demonstrate documentation of trends in vital signs and clinical information in critically ill patients
	21	Demonstrate re-triage of critically ill patients in the context of logistical limitations and operational environment

TLO	ELO	
Given an austere resuscitative surgical care (ARSC) scenario, perform management of shock in the	1	Describe management of hypovolemic shock in critically ill patients
	2	Describe management of anaphylactic shock in critically ill patients

austere environment in accordance with Defense Committees on Trauma (DCoT), Committee on Surgical Combat Casualty Care (CoSCCC) and Joint Trauma System (JTS) Guidelines	3	Describe management of septic shock in critically ill patients, IAW JTS Sepsis in PFC CPG
	4	Describe management of cardiogenic shock in critically ill patients
	5	Describe management of obstructive shock in critically ill patients, specifically the development of pulmonary embolism and tension pneumothorax
	6	Describe management of neurogenic shock in critically ill patients

TLO	ELO	
Given an austere resuscitative surgical care (ARSC) scenario, perform management of non-traumatic critical care patients in the austere environment in accordance with Defense Committees on Trauma (DCoT), Committee on Surgical Combat Casualty Care (CoSCCC) and Joint Trauma System (JTS) Guidelines	1	Describe critical care management of infections, specifically the early recognition and management of sepsis
	2	Describe critical care management of acute coronary syndrome (ACS)
	3	Describe critical care management of venous thromboembolism (VTE)
	4	Describe critical care management of acute renal failure (ARF)
	5	Describe critical care management of stroke
	6	Describe critical care management of pediatric patients
	7	Describe critical care management of local endemic infectious diseases
	8	Describe critical care management of local envenomation injuries
	9	Describe critical care management of delirium

TLO	ELO	
Given an austere resuscitative surgical care (ARSC) scenario, perform nursing care of critical care patients in the austere environment in accordance with Defense Committees on Trauma (DCoT), Committee on Surgical Combat Casualty Care (CoSCCC) and Joint	1	Describe frequency and methods of lip care
	2	Describe frequency and methods of oral/nasal care
	3	Describe frequency and methods of oral/dental care
	4	Describe frequency and methods deep breathing management/cough
	5	Describe frequency and methods of casualty repositioning and passive range of motion in a non-ambulatory casualty
	6	Identify available resources for padding a non-ambulatory casualty
	7	Demonstrate patient repositioning and use of padding for a non-ambulatory patient
	8	Identify techniques to address a casualty's psycho-social needs

Trauma System (JTS) Guidelines	9	**Describe the frequency and methods of casualty nutrition support**
	10	**Describe frequency and methods for casualty hygiene**
	11	**Describe frequency and methods for casualty bowel management**
	12	**Describe frequency and methods for IV site & catheter management**
	13	**Describe casualty Deep Vein Thrombus (DVT) prevention methods**
	14	**Describe suction of ETT using manual or improvised suction device**
	15	**Describe frequency and methods for OG/NG management**
	16	**Describe frequency and methods for foley management**
	17	**Describe frequency and methods for drain management**
	18	**Given a team scenario, demonstrate a comprehensive nursing management strategy that includes IV site management, DVT prevention, ETT suctioning, OG/NG management, foley management, and drain management IAW JTS Nursing Intervention in PFC CPG**

LEARNING OBJECTIVES

Module 9 –Patient Packaging and Transport

TLO	ELO	
Given an austere resuscitative surgical care (ARSC) scenario, perform patient packaging and transport in the austere environment in accordance with Defense Committees on Trauma (DCoT), Committee on Surgical Combat Casualty Care (CoSCCC) and Joint Trauma System (JTS) Guidelines	1	Demonstrate the ability to perform patient packaging including management and inventory of all resources for casualty packaging including patient padding, spine immobilization as indicated
	2	Demonstrate the reporting methods for communicating casualty status and interventions in preparation for casualty transport
	3	Demonstrate methods for medication preparation including sequential doses, IV fluids, and/or drip medications for transport team, including tagging/labeling of medications
	4	Demonstrate appropriate dosing and documentation of medications in advance of patient movement, including sedation, analgesia, antibiotics, vasoactive and paralytic medications
	5	Demonstrate the methods of active/passive hypothermia management for casualty transport
	6	Describe the flight stressors in preparation for casualty transport
	8	Demonstrate application of ear and eye protection prior to casualty transport
	9	Demonstrate the methods to secure patient in the litter, and lines, tubing, patient dressings and other equipment in preparation for casualty transport
	10	Demonstrate patient hand off methods during both patient receiving and patient drop-off including degraded communications environments (e.g., language barriers and loud/hectic operational environments)
	11	Describe the indications for decompression of gastric, bladder and/or chest tubes in preparation for casualty transport
	12	Describe the altitude/platform requirements for the release of residual air in IV fluid bags in preparation for casualty transport
	13	Demonstrate movement of a patient to an evacuation platform.
	14	Demonstrate appropriate use of PCC documentation tools required by Service policy
	15	Demonstrate movement of a patient to an evacuation platform while providing ongoing resuscitation and stabilization

TLO	ELO	
**Optional Section** Given an austere resuscitative surgical care (ARSC) scenario, perform en-route care in the austere environment in	1	Demonstrate ongoing sedation during transport
	2	Demonstrate urgent/emergent care of vent failure during transport
	3	Demonstrate urgent/emergent care of monitor failure during transport
	4	Demonstrate urgent/emergent care of hypotension management during transport

**accordance with Defense Committees on Trauma (DCoT), Committee on Surgical Combat Casualty Care (CoSCCC) and Joint Trauma System (JTS) Guidelines**	5	**Demonstrate urgent/emergent care of hypoxia management during transport**
	6	**Recognize and treat tension PTX during transport**
	7	**Demonstrate urgent/emergent care of tube failure during transport**
	8	**Demonstrate urgent/emergent care of line failure during transport**
	9	**Demonstrate urgent/emergent care of drain failure during transport**

**The purpose of this section is to train on dynamic patient movement, not formal patient evacuation between Roles of care. These ELOs do not replace a formal course for en-route care like the Joint En-route Care Course (JECC).*

Module 10 – Prolonged Patient Holding

TLO	ELO	
**Given an austere resuscitative surgical care (ARSC) scenario, perform patient holding in the austere environment in the austere environment in accordance with Defense Committees on Trauma (DCoT), Committee on Surgical Combat Casualty Care (CoSCCC) and Joint Trauma System (JTS) Guidelines**	1	**Describe reallocation of resources in an unanticipated patient holding scenario**
	2	**Demonstrate re-triage of patients during delayed evacuation**
	3	**Describe ethical principles and moral injury prevention when patient holding is prolonged**
	4	**Describe aspects of austere nursing care that are altered by prolonged patient holding**
	5	**Describe aspects of austere critical care that are altered by prolonged patient holding**
	6	**Describe definitive surgery – abdominal closure**
	7	**Describe definitive surgery – vascular repair**
	8	**Describe definitive surgery – repeat wound care (washouts, etc.)**
	9	**Describe definitive surgery – bowel anastomosis**
	10	**Describe indications for extubation**
	11	**Describe differences in decision making for patients that cannot be transported**
	12	**Describe alterations in team dynamics when patient evacuation is delayed**

Developed by the

JOINT TRAUMA SYSTEM
