# Supplementary material for: Fighting to Train—Implementation of a Train Like You Fight Joint Role 2 Austere Surgical Care Curriculum
Source: Mil Med. 2025 Dec 4;191(7-8):e1536–45. doi: 10.1093/milmed/usaf576 (PMC13331495; doi:10.1093/milmed/usaf576)
Supplement: usaf576_Supplementary_Data [file usaf576_supplementary_data.zip › usaf576_Supplementary_Data/SUPP TABLE 1_Role_2_Austere_Surgical_Care_Curriculum_JULY_2.docx]

**Supplemental Table 1:** Identified Austere Resuscitative Surgical Care Courses

| **ARSC/Role 2 Course** | **Service** | **Setting/ Environment** | **Teams Trained** |
| --- | --- | --- | --- |
| Emergency War Surgery Course (EWSC) CPG Didactic Curriculum | Joint | Recorded PowerPoint based lectures available through the JTS website | Joint: Standardized JTS CPG based open access curriculum. |
| EWSC – Advanced Surgical Skills for Exposure in Trauma (ASSET+) | Joint | Cadaver based surgical skills course | Required every three years for all military general surgeons; surgical technicians routinely participate |
| EWSC – Combat Orthopedic Trauma Course (COTS+) | Joint | Cadaver based surgical skills course | For individual orthopedic surgeons; surgical technicians routinely participate |
| Definitive Surgical Trauma Care (DSTC) Course | Civilian | International Organization: International Association for Trauma Surgery and Intensive Care (IATSIC) | Not Team Based. Primarily designed for surgeons, anesthesia providers incorporated. |
| Strategic Readiness Center (STaRC) Course | Army | Military trauma center clinical rotations, cadaver and simulation- based field exercises | Army Forward Resuscitative Surgical Detachment (FRSD); Navy Fleet Surgical Team (FST) |
| Army Trauma Training Center (ATTC) Course | Army | Military-Civilian Partnership (MCP). Includes trauma clinical rotations, didactics and team-based simulation. | FRSD |
| Navy Trauma Training Center (NTTC) Course | Navy | MCP. Includes trauma clinical rotations, didactics and team-based simulation and cadaver training. | Navy Expeditionary Resuscitative Surgical System (ERSS); Various Navy Role 2 teams |
| ERSS Role 2 Light Maneuver Course | Navy | Field Exercises; Team high fidelity simulation training using a human or mannequin worn partial task surgical simulation or “cut suit” ( © STOPS MED, San Diego, CA) in a variety of austere environments | Primarily ERSS. U.S. Marine Corps (USMC) Role 2 teams have intermittently taken course |
| 1^st^ Medical Battalion USMC Role 2 Certification Training | USMC | Low fidelity simulation team training | USMC Role 2 |
| Shipboard Surgical Trauma Training (S2T2) Course | Navy | Independent Civilian Contractor; High fidelity simulation training mimicking shipboard environment | FST |
| Ground Surgical Team (GST) Course | Air Force | Service Only. Independent Civilian Contractor; High fidelity simulation training | GST |
| NATO Special Operations Surgical Team (SOST) Course | NATO | NATO; High fidelity simulation; | Special Operations Forces Surgical Teams |
