# Supplementary material for: Fighting to Train—Implementation of a Train Like You Fight Joint Role 2 Austere Surgical Care Curriculum
Source: Mil Med. 2025 Dec 4;191(7-8):e1536–45. doi: 10.1093/milmed/usaf576 (PMC13331495; doi:10.1093/milmed/usaf576)
Supplement: usaf576_Supplementary_Data [file usaf576_supplementary_data.zip › usaf576_Supplementary_Data/Supplemental Box 1 (new).docx]

Supplemental Box 1: Description of Work Group (WG) Curriculum Development Process

| 1. **Composition of the WG:** The WG comprised a multidisciplinary team of Department of Defense (DoD) trauma experts, including trauma surgeons, emergency physicians, anesthesiologists, critical care specialists, nurses, and non-commissioned officers (NCOs). All members brought significant operational experience from deployed settings, ensuring the curriculum reflected real-world austere conditions. 2. **Structure and Function of Meetings:** The 24 meetings were held biweekly over 14 months, each lasting approximately 2 hours, and were facilitated by a designated WG chair with expertise in trauma surgery and curriculum development. Meetings were conducted virtually, ensuring broad participation from geographically dispersed DoD experts. The agenda included reviewing existing curricula, discussing operational challenges, and refining best practices, with each session building on prior discussions to maintain an iterative approach. 3. **Facilitation:** Facilitation involved a structured format led by the WG chair, supported by a dedicated note-taker. The chair guided discussions to ensure all voices were heard, managed time allocation for agenda items, and resolved conflicts through mediation. Subgroup assignments were made post-meeting to address specific topics, with subgroups reporting back in subsequent sessions. 4. **Frameworks and Evaluation Criteria:** The WG assessed existing curricula using a framework based on DoD and Joint Trauma System (JTS) standards, focusing on criteria such as relevance to austere environments, operational applicability, and alignment with DoD trauma care guidelines. A scoring rubric was applied to evaluate content, feasibility, and evidence base, with scores averaged across WG members to prioritize high-performing elements for inclusion. 5. **Consensus-Building Techniques:** Decisions were made using a combination of subgroup analysis, voting, and subject matter expert (SME) consensus. Subgroups analyzed specific curriculum components and presented findings. Voting was conducted via anonymous polls on key decision, requiring a 70% majority for approval. SME consensus was achieved through open discussion, with the chair synthesizing input to resolve remaining disagreements, ensuring all perspectives—especially from operational experts—were integrated. 6. **Recording Decisions:** Minutes were meticulously recorded for every meeting by a designated note-taker, capturing attendance, agenda items, decisions, and the rationale behind them. These minutes were shared with the WG within 48 hours for review and approval. Convergence on best practices was achieved through iterative refinement, with recurring themes tracked across meetings and validated against operational experience and JTS data, culminating in a finalized curriculum draft by the 24th meeting. 7. **Committee on Surgical Combat Casualty Care (CoSCCC) Review and Approval:** Drafts of the Joint Role 2/Austere Resuscitative Surgical Care (R2/ARSC) modules, terminal learning objectives and enabling learning objectives was presented at the March and August 2023 CoSCCC meetings; CoSCCC membership was given an opportunity to review the curriculum and provide input. After CoSCCC input, the WG voted on and approved the Joint R2/ARSC curriculum in September 2023. In February 2024, the Joint R2/ARSC curriculum modules were sent out to CoSCCC membership via email. CoSCCC membership voted electronically and the Joint R2/ARSC curriculum was approved. |
| --- |
